# 3D-Integrated metasurfaces for full-colour holography

**DOI:** 10.1038/s41377-019-0198-y

**Published:** 2019-09-18

**Authors:** Yueqiang Hu, Xuhao Luo, Yiqin Chen, Qing Liu, Xin Li, Yasi Wang, Na Liu, Huigao Duan

**Affiliations:** 1 State Key Laboratory of Advanced Design and Manufacturing for Vehicle Body, College of Mechanical and Vehicle Engineering, Hunan University, 410082 Changsha, People’s Republic of China; 2 Kirchhoff Institute for Physics, University of Heidelberg, Im Neuenheimer Feld 227, 69120 Heidelberg, Germany

**Keywords:** Metamaterials, Nanophotonics and plasmonics

## Abstract

Metasurfaces enable the design of optical elements by engineering the wavefront of light at the subwavelength scale. Due to their ultrathin and compact characteristics, metasurfaces possess great potential to integrate multiple functions in optoelectronic systems for optical device miniaturisation. However, current research based on multiplexing in the 2D plane has not fully utilised the capabilities of metasurfaces for multi-tasking applications. Here, we demonstrate a 3D-integrated metasurface device by stacking a hologram metasurface on a monolithic Fabry–Pérot cavity-based colour filter microarray to simultaneously achieve low-crosstalk, polarisation-independent, high-efficiency, full-colour holography, and microprint. The dual functions of the device outline a novel scheme for data recording, security encryption, colour displays, and information processing. Our 3D integration concept can be extended to achieve multi-tasking flat optical systems by including a variety of functional metasurface layers, such as polarizers, metalenses, and others.

## Metasurfaces: Stacking up for impressive optics

A compact optical device comprising vertically stacked metasurfaces can simultaneously generate microscopic text and full-colour holograms for encrypted data storage and colour displays. Metasurfaces are ultrathin, synthetic materials patterned with features smaller than the wavelength of light, enabling researchers to manipulate how light rays behave when they hit the surface. Huigao Duan and co-workers at Hunan University in Changsha, China and the University of Heidelberg in Germany fabricated a metasurface device that simultaneously generates full-colour holograms and microprint – tiny lettering - for use in encryption and security. Their design comprises stacked metasurfaces - one that generates colour microprint when illuminated with white light, and another that projects holograms in full colour under RGB lasers. The device can encrypt and store data more efficiently, and with lower levels of crosstalk, than previous designs.

## Introduction

Metasurfaces open a new paradigm to design optical elements by shaping the wavefront of electromagnetic waves by tailoring the size, shape, and arrangement of subwavelength structures^1–3^. A variety of metasurface-based devices, such as lenses^4–8^, polarisation converters^9^, holograms^10–12^, and orbital angular momentum (OAM) generators^13–15^, have been demonstrated. The performance of metasurface-based devices can even surpass that of conventional refractive elements because more extreme, arbitrary, and multiplexing wavefronts can be modulated in subwavelength pixels.

Among the various advantages of metasurface-based devices, the most fascinating property is their capability to achieve flat and ultrathin optical elements, which offers a great opportunity to realise compact optical devices with multiple functions via two-dimensional (2D) and three-dimensional (3D) integration of discrete metasurfaces. For example, anisotropic structures or interleaved subarrays have been specifically arranged in a 2D plane to obtain multiplexed devices^10,16–18^. To reduce crosstalk, plasmonic effects^19,20^, Pancharatnam–Berry phase (PB phase) conversion efficiency selectivity^10,18^, multiple angle off-axis illumination^16^, and chromatic aberration^21^ have been demonstrated. However, shortcomings such as the low efficiency of plasmonic nanostructures, incidence polarisation requirements, large crosstalk, extra angle freedom, and complexity of readout of the Fresnel hologram result in degraded performance for multi-wavelength and broadband optical devices. In addition, 2D multifunction integration has the bottlenecks of limited design freedom due to space constraints. Compared to 2D integration, 3D stacking of metasurface-based devices with different functions in the vertical direction can combine the advantages of each device, reduce the processing difficulty of each device, increase design freedom, and generate new features to improve the integration of optical devices and achieve compact multifunctional devices.

Vertically stacked metasurfaces for potential 3D-integrated optical devices have attracted much attention. Various devices, such as wavelength multiplexed lenses^22,23^, monochromatic aberration correctors^24,25^, and retroreflectors^26^, have been demonstrated. Among them, the wavelength-multiplexed lens has been achieved by stacking a couple of monochromatic lenses with multiple overlay fabrication processes. Its principle is essentially the same as that of the interleaved subarrays in the 2D plane, but this device allows for the utilisation of comparatively simple structures in each lens, thus enabling more freedom of design and a wider process window in fabrication. The monochromatic aberrations corrector and retroreflector, obtained by combining two metasurfaces with different functionalities, can achieve a more complicated function, which is similar to the concept of a compound lens in current optical systems but greatly reduces the size of the device. These examples have demonstrated the advantages of ultrathin and flat metasurfaces for 3D stacking and show great potential for future compact optics applications. However, most of the current research has focused on 3D integration of metalenses and has not fully utilised the capabilities of metasurfaces for multi-tasking applications. Very recently, an attempt has been made to achieve holographic colour prints based on 3D-printed stacked microscale diffractive optics and nanostructured colour filters^27^, but this approach suffers from a small field-of-view (FOV), multiple diffraction orders, low data density, relatively large crosstalk, and limited fabrication accuracy, leading to a great challenge for full-colour holography applications.

In this work, we demonstrate using 3D integration of metasurfaces to realise full-colour holography by stacking a monolithic colour filter microarray and hologram metasurface. Such an integrated device provides a possible solution to solve the bottleneck problems (e.g., large crosstalk, small FOV, extra angle freedom, and strict polarisation requirements) of full-colour holography. Simultaneously, the function of microprints enabled by a monolithic colour filter microarray are still maintained for various multifunction applications. A colour microprint image is observed when the device is illuminated by white light, while a full-colour hologram image can be projected into the far field under red (R), green (G), and blue (B) laser illumination by mixing three independent greyscale hologram images. Compared to the existing approaches for full-colour holography, which have been demonstrated with plasmonic nanostructures and dielectric nanostructures, the current device based on 3D-integrated metasurfaces has the advantages of low crosstalk, polarisation independence, high efficiency, and simple fabrication process. Our work utilising the thinness and flatness of metasurfaces to build an integrated device that surpasses the performance and functions of traditional optical devices presents an important example demonstrating the uniqueness and advantages of metasurfaces for 3D integration and represents substantial progress in exploring the irreplaceable applications of metasurfaces. The concept of 3D-integrated metasurfaces could be extendable by stacking more metasurfaces, such as polarizers and metalenses, in the vertical direction to form other multifunctional ultrathin optical systems.

## Results

### Design and fabrication of a 3D-integrated full-colour metasurface

**Fig. 1:**
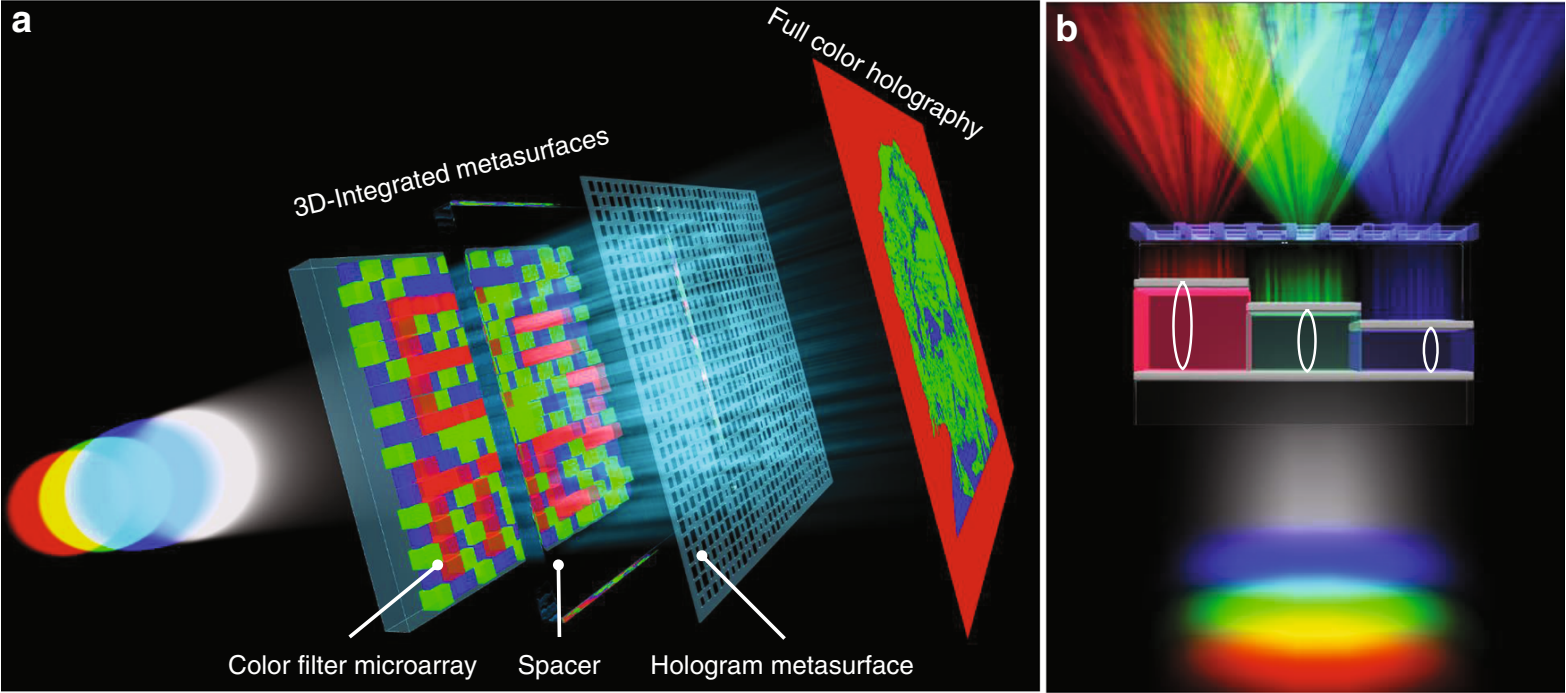
Schematic of the 3D-integrated metasurfaces for full-colour holography by vertically stacking a colour-filter microarray with a hologram metasurface. **a** Exploded view of the 3D-integrated metasurfaces. The colour-filter microarray can be specifically arranged to form a colour microprint under white-light illumination (e.g., a mass-energy equation image), while the hologram metasurface can encode hologram information. When the red (R), green (G), and blue (B) lasers are illuminated simultaneously, three independent hologram images are generated in the far field. By combining the three holograms, an arbitrary full-colour hologram image can be obtained (e.g., a portrait of Albert Einstein). **b** Front view of three micro-units of the 3D-integrated metasurfaces. The colour filters consist of metal/dielectric/metal Fabry–Pérot (MDMFP) cavity resonators. When the device is illuminated by red (R), green (G), and blue (B) lasers, the light can only go through the filers with the closest resonance wavelength compared to the source lasers and subsequently shine on the hologram metasurface, generating three independent far-field monochromatic greyscale hologram images

Figure 1 illustrates the schematic diagram of the 3D-integrated metasurface that is composed of microscale stepwise structures and a hologram metasurface for full-colour holography. The microscale stepwise structures consist of an array of metal/dielectric/metal Fabry–Pérot (MDMFP) cavity resonators, which act as colour filters, with varied dielectric thicknesses. MDMFP colour filters are proven to have high transmission efficiency, wide colour gamut, and narrow spectral linewidth compared to that of plasmonic colour filters^28–30^. The hologram metasurface is composed of isotropic dielectric nanostructures, which can manipulate the propagation phase of light at the subwavelength scale and generate high-quality far-field hologram images. As Fig. 1b shows, when the device is illuminated by RGB lasers, the lasers only go through the filters with their closest resonance wavelengths and subsequently shine on the hologram metasurface, generating three independent far-field monochromatic greyscale hologram images. By carefully mixing RGB channels, a full-colour hologram image can be achieved. The metasurface is carefully designed to form a projection at the desired wavelength and encode the holographic information on the specifically arranged colour filters, including the colour microprint information. Compared to existing metasurface-based colour holography using the wavelength multiplexing concept with plasmonic and PB phase-based nanostructures, the current design of integrating a colour filter microarray with a hologram metasurface has several advantages. First, the FP cavity colour filters and the dielectric metasurface ensure higher transmission and diffraction efficiency for far-field holography than those of transmissive plasmonic holography. Second, due to the narrower resonances of FP colour filters, the smaller crosstalk of different channels enables higher quality full colour holography. Third, the device is polarisation independent compared to the colour holography achieved by a PB phase-based metasurface, which requires a circular incident polarisation. Finally, the device can be simply fabricated based on common electron-beam lithography (EBL) and metal evaporation processes without involving any pattern transfer process.

**Fig. 2:**
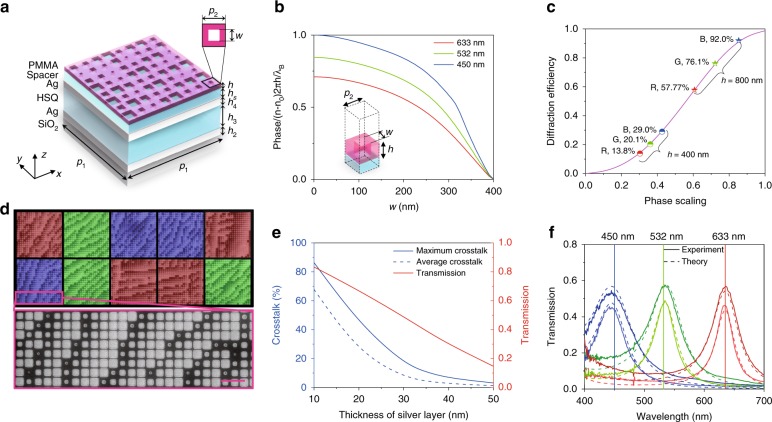
Design and fabrication of 3D-integrated metasurfaces. **a** Schematic of a unit cell of the 3D-integrated metasurfaces. **b** The phase change normalised based on the B channel with varied sizes of nanoholes. **c** The diffraction efficiency with different phase scaling and B, G, and R efficiency of PMMA with a refractive index of 1.48 in the visible light range at 400 nm (29.0%, 20.1%, and 13.8%) and 800 nm (92.0%, 76.1%, and 57.8%, respectively) height. **d** False-colour SEM image of the device with a colour filter microarray (unit size: 10 μm) and hologram metasurface (structure period: 400 nm). Different colours represent the separated colour filters in the device. Scale bar: 1 μm. **e** Effect of silver film thickness (the thickness of the two silver films is equal) on maximum crosstalk, average crosstalk and transmission efficiency. Increasing the silver film thickness can suppress crosstalk, but it also reduces transmission efficiency. **f** Theoretical and experimental transmission spectra for R (633 nm), G (532 nm), and B (450 nm) channels of 26-nm-thick silver layer colour filters (dark-colour serial line) and 31-nm-thick silver layer colour filters (light-colour serial line)

Figure 2a shows a more detailed schematic of a single unit of the device. The upper part shows the hologram metasurface composed of dielectric nanoholes, which are proven to be mechanically more stable than nanopillars in a high aspect ratio. The phase accumulation is realised by a propagation phase of *φ* = *n*_eff_*k*_0_*h*, where *n*_eff_ is the effective refractive index of the nanostructures and *k*_0_ and *h* represent the propagation wave vector in air and the height of the nanostructures, respectively. By altering the size (*w*, determining the value of *n*_eff_) of the nanoholes, different phase responses can be obtained to shape the desired wavefront for the hologram. In this work, we applied the same height and period (400 nm) of nanoholes for R (633 nm), G (532 nm), and B (450 nm) wavelengths, which allows one-step fabrication of metasurfaces for different wavelengths. Figure 2b shows the phase change normalised based on the B channel with varied sizes of nanoholes analysed by the finite difference time domain (FDTD) method. In principle, an approximately continuous phase can be selected by this curve. In Supplementary section 1, we have proven that more phase levels can achieve higher diffraction efficiency and image quality. However, the increase is not obvious when the phase levels are greater than 8. In our device, 16 phase levels were selected considering the trade-off between the diffraction efficiency and fabrication difficulty. In addition, the scaling of the phase will only reduce the efficiency of the holography without affecting the hologram information (Supplementary section 1). Figure 2c shows the diffraction efficiency with different phase scaling and the B, G, and R efficiency of PMMA with a refractive index of 1.48 in the visible-light range at heights of 400 nm (29.0%, 20.1%, and 13.8%) and 800 nm (92.0%, 76.1%, and 57.8%, respectively). Here, to demonstrate the device function and reduce fabrication difficulty, PMMA with a height of 400 nm was used. However, higher structures and materials with a larger refractive index can be applied to achieve higher diffraction efficiency.

**Fig. 3:**
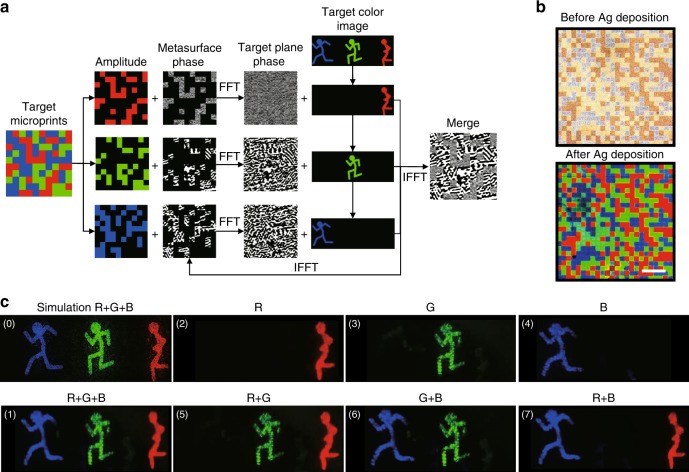
Concept demonstration of the 3D-integrated metasurfaces. **a** The flowchart of the modified Gerchberg–Saxton (GS) algorithm to generate the hologram phase. FFT is the fast Fourier transform, and IFFT is the inverse fast Fourier transform. **b** Transmission images of a random trichromatic colour microprint with 24 × 24 pixels captured by an optical microscope: before (top) and after (bottom) the deposition of the top silver layer. The scale bar is 50 μm. **c** The far-field hologram images of “running man” captured by a digital camera on a screen: (0) the simulation result of R + G + B channels; (1)–(7) seven channels achieved by combining the RGB in the experiments

The basic configuration of the colour filter is an Ag/hydrogen silsesquioxane (HSQ)/Ag resonance cavity structure on a quartz substrate. Silver layers act as semi-reflective films. HSQ is an inorganic negative-tone electron-beam resist that converts to SiO_*x*_ thin film as a dielectric layer under electron exposure. The different transmissive colours are achieved by an FP cavity effect, which means that different resonance wavelengths can be obtained due to the constructive interferences when altering the thickness of the dielectric layer. Through analytical analyses with multilayer film theory^31^, we found that the thickness of the dielectric layer only changes the position of the peak and has a limited effect on transmission efficiency, and a thicker film leads to multiple orders of peaks (Fig. S3b). For the RGB wavelengths used in the experiment, we calculated the influence of the silver film thickness (the thicknesses of the two silver films are equal) on the maximum crosstalk, average crosstalk and transmission efficiency, as shown in Figs. 3e and S3c, d. Increasing the silver film thickness can suppress crosstalk, but it also reduces transmission efficiency. When the silver film is thicker than 30 nm, the crosstalk reduction is not obvious.

The whole device was mainly created by two EBL processes: the greyscale EBL process for MDMFP colour filters and an overlay EBL process for a hologram metasurface. Details of the fabrication processes and parameters can be found in the section “Methods” and Supplementary section 4. The size of the colour filter (*p*_1_) can be varied greatly, but the plasmonic effect is exhibited when it is less than the wavelength. In previous work, we achieved a minimum size of 500 nm for a colour filter^28^. Here, *p*_1_ was set as 10 μm. To match the RGB wavelengths in our experiments, the colour palette was first fabricated (see Supplementary section 5). The size of each block was 10 μm, which was the same as the period of the colour filter microarray. The transmission spectra and heights of the blocks were measured by a spectrometer and atomic force microscopy (AFM) to determine the relationship between the exposure dose and the height (Fig. S5c). With such a relationship, a colour filter microarray with a designed colour distribution can be fabricated. A 150-nm-thick HSQ spacer was then coated to flatten the surface of the colour filter microarray and protect the silver layer from oxidation. Note that the upper dielectric layers on the colour filter microarray act as an antireflection layer and have a negligible effect on the performance of the colour filter (Fig. S6a, b). Figure 2d shows the false-colour scanning electron microscopy (SEM) image of the fabrication result of the 3D-integrated metasurface device. Colour blocks were added to show the separated different colour filters. The enlarged view shows the detailed metasurface structure. As we have shown above, there is a balance between the transmission efficiency and crosstalk. In the following experiments, two kinds of devices with silver layers with different thicknesses, 26 and 31 nm, were fabricated. Figure 2f shows the comparison between the experimental transmission spectra for the RGB channels and the theoretical calculated spectra based on the corresponding measured height of the blocks. The corresponding structure heights of the three spectra were 93, 126, and 161 nm for the 26-nm-thick silver layer devices (dark-colour serial lines) and 95, 128, and 164 nm for the 31-nm-thick silver layer devices (light-colour serial lines). We can see that the results match well between the experiment and the theory. Due to fabrication inaccuracy, the crosstalk in the experiment is larger than the theoretical prediction. However, the average crosstalk of the 31-nm-thick silver devices (6.1% in theory and 9.3% in the experiments) is still significantly less than that of the 26-nm-thick silver devices (11.4% in theory and 17.5% in the experiments).

### Concept demonstration

The Gerchberg–Saxton (GS) algorithm is an iterative algorithm often applied for computer-generated holography (CGH) to retrieve the phase distribution of a hologram. To achieve the dual functions of microprint and holography, we developed a modified GS algorithm for encoding two types of independent information into a microprint and hologram. The flowchart of the algorithm is shown in Fig. 3a. First, each pixel of the colour image was matched by the closest colour in the palette. In this way, the height and the exposure dose of the FP cavity were obtained by the palette database, which was then used in the greyscale EBL. The colour image was then recoloured accordingly and separated into different colour components. Suitable colour filters for RGB channels with small crosstalk between each other were selected. The corresponding normalised amplitude distribution was also generated based on the transmittance of each pixel at the RGB wavelengths. Subsequently, the amplitude distribution was input into the main body of the modified GS algorithm to calculate the hologram phase to achieve the separated R, G, and B greyscale hologram images. Due to the chromatic aberration of the metasurface, the target hologram image projected by laser of *λ*_*j*_ should be scaled by the ratio of $\frac{{\lambda _i}}{{\lambda _j}}$ to match the size of the image projected by a laser of *λ*_*i*_ in the screen to ensure accurate colour mixing (Supplementary section 2). In addition, the target hologram image was pre-compensated for each wavelength to eliminate the distortion in the far field due to the large field of view (FOV), which is up to 76.9° × 76.9° for 633 nm (Supplementary section 2). To eliminate zero-order light, the hologram image was reconstructed in a certain quadrant with zero-order light as the origin (Fig. S2b). Finally, the three-phase distribution components were merged together to generate the final hologram phase.

To verify the concept, a 3D-integrated metasurface with a random trichromatic colour microprint and “running man” colour holography was demonstrated. Figure 3b shows the transmission optical micrographs of 24 × 24 pixels (i.e., 240 μm × 240 μm) before (top) and after (bottom) the deposition of the top 31-nm-thick silver layer (see the section “Materials and methods” for optical characterisation methods). We can see that three vivid colours were generated after the formation of the FP cavities. Then, the spacer layer and hologram metasurface were fabricated on the colour microprint. The hologram image was captured on the far-field screen by RGB laser illumination. The experimental setup for the hologram characterisation can be found in Fig. S7. Because of the polarisation-independent property of the device, no waveplates for polarisation conversion are needed in the setup. Figure 3c shows the hologram images of seven different channels by changing the input channels of the lasers. The comparison between the simulation and experiment shows that the device recovers the designed image information well. The diffraction efficiency for the independent RGB channels in the experiments is 10.8%, 12.6%, and 22.1%, respectively. Therefore, the multiple functions of a microprint and wavelength multiplexed holograms can be used for various types of encryption to enhance information security. In addition, different combinations of laser channels enable us to create a full-colour image by balancing the input powers of RGB.

### Encryption and full-colour holography

#### Encryption

**Fig. 4:**
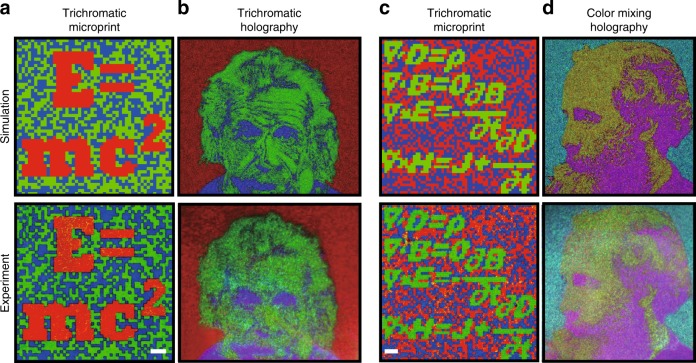
Two 3D-integrated metasurfaces for optical encryption: (top row) simulations and (bottom row) experiments. **a** Mass-energy equation in the trichromatic microprint with 50 × 50 pixels. **b** Trichromatic portrait of Albert Einstein in a hologram image. **c** Maxwell equations in the trichromatic microprint with 60 × 60 pixels. **d** Mixed colour portrait of Jams Clerk Maxwell in the hologram image, demonstrating the combination capability of RGB channels to obtain yellow, purple, and cyan colours. The scale bars in **a** and **c** are 50 μm

Figure 4 shows two encryption devices based on the dual function of the microprint and colour meta-hologram. Figure 4a shows the simulated (top) and experimental (bottom) trichromatic microprint results of the first device, including the information of the mass-energy equation proposed by Albert Einstein. The main information is displayed in red, and the background is the random green and blue colour. The microprint includes 50 × 50 pixels (i.e., 500 μm× 500 μm). The experimental result of the final microprint with a dielectric spacer and metasurface layers on it is almost identical to the designed image, which proves that the upper layers do not affect the function of the colour-filter microprints (Fig. S6c, d shows the microprints without upper layers). The far-field hologram image projected from the 1250 × 1250 meta-units was captured by RGB laser illumination (Fig. 4b). The hologram image projected a modified trichromatic portrait of Albert Einstein, which was a combination of the RGB binary images. The experimental hologram image retrieved most of the details of the photos, including the eyes, nose, and even wrinkles and hair. The concept was also applied to obtain a mixed colour hologram. Figure 4c, d show the microprint of the Maxwell equations and a holographic portrait of James Clerk Maxwell, respectively. The Maxwell equations consisted of 60 × 60 pixels (i.e., 600 μm × 600 μm). The yellow, purple, and cyan colours were achieved by a combination of binary images of R and G, R and B, G and B, respectively, indicating that the overlap among the RGB channels was well designed and achieved.

#### Full-colour holography

**Fig. 5:**
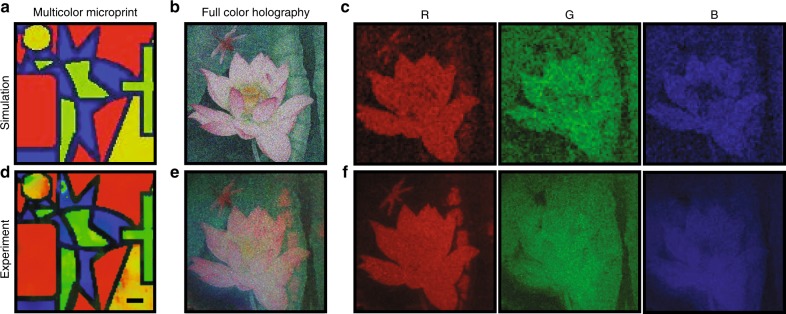
Full-colour holography demonstration with 3D-integrated metasurfaces device. **a** Simulated “four colour theorem” painting consisting of five different colours (including the boundary), which are red, green, yellow, blue, and navy blue, respectively. **b** Simulated “Chinese painting of lotus” hologram image, consisting of a pink lotus with yellow flower core, green lotus leaf, dark water, and a red dragonfly. **c** The greyscale images of the RGB components of the painting. **d** The fabricated colour filters containing five colours with 100 × 100 pixels. The scale bar is 100 μm. **e** The hologram image projection in the experiment combining the RGB channels and **f** its corresponding RGB components

By fabricating MDMFP cavities with different dielectric thicknesses in the integrated metasurface device, a full-colour microprint of an arbitrary image could be realised. In addition, full-colour holography can be obtained by combining greyscale monochrome images of RGB channels. As an example, a full-colour 3D-integrated metasurface holography device is shown in Fig. 5. The colour-filter microprint was designed as a part of a “four colour theorem” painting. The painting shown in Fig. 5a includes five different colours (including the boundary), which are red, green, yellow, blue, and navy blue. First, the closest colours were matched in the palette and then fabricated with the corresponding exposure dose by greyscale EBL. The experimental colour filters shown in Fig. 5d consist of 100 × 100 pixels (i.e., 1000 μm × 1000 μm) and reproduced the painting colours well. To minimise the crosstalk between RGB hologram images, red, green, blue, and navy-blue filters were selected as the working filters. The spectra are shown in Fig. S8. Then, the metasurface encoded with a full-colour image with greyscale information of a “Chinese painting of lotus” was fabricated to demonstrate full-colour holography, as shown in Fig. 5b. The painting consists of a pink lotus with a yellow flower core, a dark green lotus leaf, dark water and a red dragonfly with light red wings. The designed painting was first separated into RGB components as the target hologram images for the experimental RGB channels, as shown in Fig. 5b. The three monochrome components consist of the greyscale information of the painting. The experimentally captured hologram image containing three monochromic parts is shown in Fig. 5e. The power of three lasers in the experiment was carefully adjusted by the polarizers to achieve the closest result to the original image. The corresponding RGB components were also captured by separately illuminating the RGB lasers on the device, as shown in Fig. 5f. Due to the small crosstalk of different channels, the hologram image recovered most details and the colours of the painting by combining the three monochrome components. The greyscale information for each single channel in Fig. 5f clearly demonstrates the information recovery ability of the hologram metasurface.

## Discussion

The performance of the device could be further enhanced with optimised structural parameters according to practical applications. For example, our control experiments prove that an appropriate silver layer is helpful for obtaining a high-quality colour hologram (Supplementary section 9). In addition, higher efficiency and less crosstalk can be achieved by utilising higher order peaks with thicker dielectric layers and suitable wavelengths (Fig. S2e). The demonstrated 3D-integration concept is also extendable for achieving more complicated functions. For example, one can choose more types of colour filters to generate a full-colour transmission image and smaller unit size to store more data because our algorithm can flexibly design the metasurface based on the distribution and size of the colour filters. Furthermore, the nanostructured hologram metasurface can introduce more complex structures, such as high-aspect-ratio and asymmetrical nanostructures, to achieve higher quality hologram images and polarisation multiplexed metasurfaces. For practical large-area and low-cost applications, the device can be mass-produced by the CMOS process, high-precision laser direct writing or nanoimprinting.

In summary, we have proposed and demonstrated a 3D-integrated metasurface concept to realise full-colour holography by vertically stacking a colour filter microarray and a nanostructured hologram metasurface. Before its integration, the device has dual functions for information encryption and storage. Miniaturised colour microprints can be obtained under white-light illumination, and full-colour holograms can be projected under appropriate RGB laser illumination. Compared to previous work on metasurface-based colour holography, the current integrated device has the advantages of low crosstalk, high efficiency, polarisation independence and simple structural configuration to achieve full-colour holography. Our work provides an excellent example of utilising the flatness and thinness of metasurfaces for 3D integration to achieve compact devices with new functions, demonstrating the great application potential of metasurfaces in multifunctional on-chip optoelectronic devices to miniaturise optical systems.

## Materials and methods

### Fabrication of the samples

First, the SiO_2_ substrate was evaporated with a silver layer of 30 nm using thermal evaporation at a rate of 0.3 Ȧ/s. Then, a 300-nm polymethyl methacrylate (PMMA) electron-beam resist layer was spin-coated. The sample was then exposed through EBL with a 30-kV voltage and a beam current of 272 pA. The exposure dose of 300 μC/cm^2^ was used to expose the marks for the overlay process. Then, 30-nm-thick gold was evaporated. The lift-off process of the sample was performed in a solution of N-methyl pyrrolidone (NMP). After the fabrication of gold marks, 200-nm HSQ layer was spin-coated on the sample as a negative-tone resist. By carefully controlling the exposure doses, we could control the thickness of the patterned HSQ resist after development. The exposure dose was determined according to the colour microprint image and the palette database in Supplementary section 4. Subsequently, a 30-nm-thick silver film top layer was evaporated again to form stepwise FP cavity resonators. The spacer was then fabricated by exposing a 200-nm HSQ layer to protect the colour filters. Finally, a layer of 350-nm PMMA was spin-coated onto the sample and then exposed to an overlay EBL process to define the binary nanostructures.

### Optical characterisation

For the optical characterisation of the microprints of the 3D-integrated metasurfaces, we used an optical microscope (Carl-Zeiss AXIO-10) with ×5 (0.13NA), ×10 (0.25NA), ×50 (0.75NA), and ×100 (0.85NA) objective lenses. The different magnifications of the devices at each process step were captured with an incoherent white-light source under transmission mode. The microprint display performance was optimised by modifying the exposure time and contrast of the CCD. Then, the fabricated integrated devices were characterised for hologram projection by the experimental setup shown in Fig. S7. The three laser diodes emitting at 450 nm (Thorlabs CPS450), 532 nm (Thorlabs CPS532), and 633 nm (Thorlabs CPS633S) were exploited to generate R, G, and B channels. The polarizers were used to manipulate the source power to achieve a suitable RGB component ratio due to the polarisation-independent property of the proposed metasurfaces. Then, two dichroic lenses (DMLP567T and DMLP490T) were used to combine the three lasers. The hologram images were projected on a screen and captured by an SLR camera. To characterise the diffraction efficiency for each wavelength, we put an optical power metre in position 1 and position 2 as Fig. S7b shows to measure the transmission power and the diffraction power of the hologram image.

## Supplementary information

Supplementary Information_Light
