# Supplementary material for: 3D-Integrated metasurfaces for full-colour holography
Source: Light Sci Appl. 2019 Sep 18;8:86. doi: 10.1038/s41377-019-0198-y (PMC6804690; doi:10.1038/s41377-019-0198-y)
Supplement: Supplementary file 1 — Supplementary Information_Light [file 41377_2019_198_MOESM1_ESM.docx]

3D-Integrated Metasurfaces for Full-Color Holography

Yueqiang Hu^1^^†^, Xuhao Luo^1†^, Yiqin Chen^1^, Qing Liu^1^, Xin Li^1^, Yasi Wang^1^, Na Liu^2^，Huigao Duan^1^*

^1^State Key Laboratory of Advanced Design and Manufacturing for Vehicle Body, College of Mechanical and Vehicle Engineering, Hunan University, Changsha 410082, People’s Republic of China

^2^Kirchhoff Institute for Physics, University of Heidelberg, Im Neuenheimer Feld 227, 69120 Heidelberg, Germany

^†^These authors contributed equally to this work

*Corresponding author. Email: duanhg@hnu.edu.cn

Supplementary information

**Section 1. Theoretical calculation of hologram metasurface**


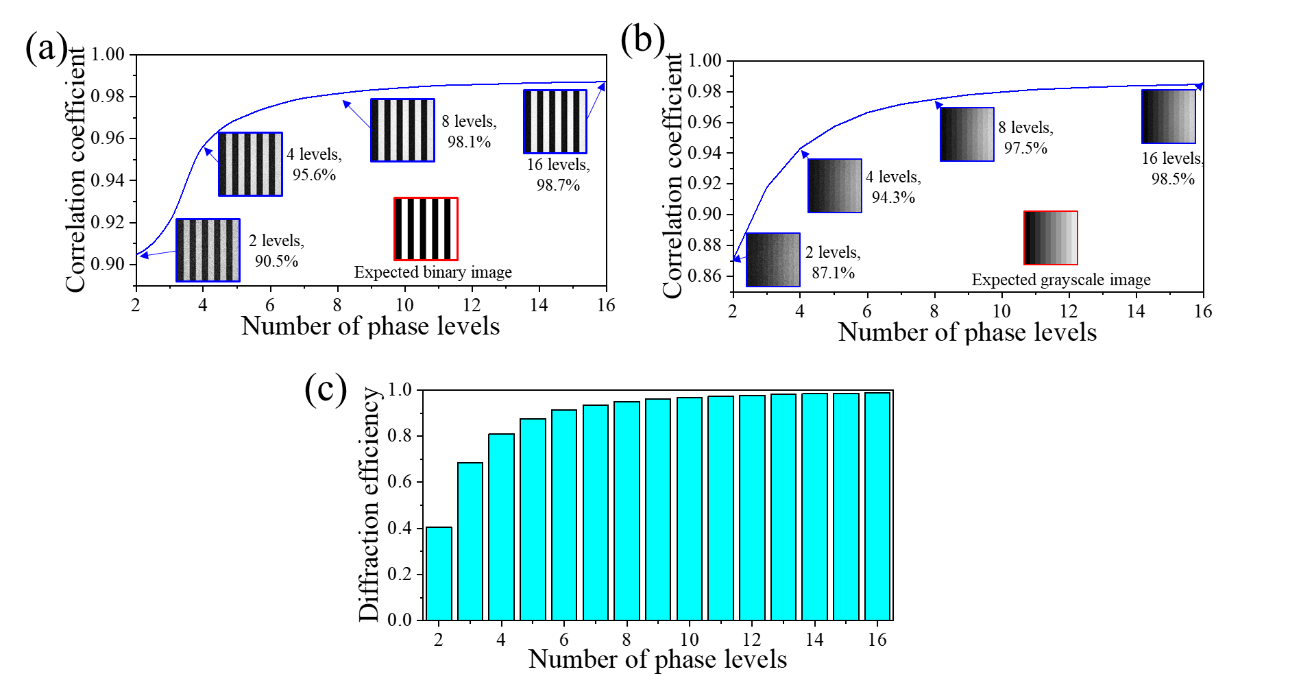


**Figure S1. Hologram theoretical calculation.** Correlation coefficient of (a) the binary image and (b) the greyscale image reconstructed by the hologram metasurface with different numbers of phase levels. The inset images correspond to the designed images (red box) and the reconstructed images (blue boxes), respectively. (c) The diffraction efficiency with different numbers of phase levels.

As described in the main text, the hologram metasurface is the isotropic square structure. By varying the size of the nanostructures, different phase response can be obtained to shape the desired wavefront for hologram. Therefore, if the period $p$ is fixed, the phase profile $\varphi\left( x,y \right)$of the nanostructures is determined by the size of the structures. The E field distribution of transmission wave can be expressed by $g\left( x,y \right)=e^{i\varphi\left( x,y \right)}$. The diffraction field in far field can be calculated by Fraunhofer integral model,

$u\left( \xi,\zeta\right)=\frac{1}{i\lambda z}e^{ik\left( z+\frac{\xi^{2}+\zeta^{2}}{2z} \right)}\int_{-\infty}^{\infty} \int_{-\infty}^{\infty} g(x,y)\cdot e^{-\frac{ik}{z}\left( \xi x+\zeta y \right)}dxdy$ (S1)

The intensity of the E field is

$|u\left( \xi,\zeta\right)|=\left| \frac{1}{i\lambda z} \right|\left| e^{ik\left( z+\frac{\xi^{2}+\zeta^{2}}{2z} \right)} \right|\left| \int_{-\infty}^{\infty} \int_{-\infty}^{\infty} e^{i\varphi\left( x,y \right)}\cdot e^{-\frac{i2\pi}{\lambda z}\left( \xi x+\zeta y \right)}dxdy \right|$ (S2)

To compare the image recovery performance of nanostructures with different numbers of phase levels, we simulate the binary image and the greyscale image recovery results with the GS algorithm. The correlation coefficient is defined to evaluate the identity between the designed image $A$ and recovery image $B$.

$corr=\frac{\sum_{m} \sum_{n} {(A}_{mn}-\bar{A})(B_{mn}-\bar{B})}{\sqrt{(\sum_{m} \sum_{n} \left( A_{mn}-\bar{A} \right)^{2})}(\sum_{m} \sum_{n} \left( B_{mn}-\bar{B} \right)^{2})}$ (S3)

The results of the binary image and greyscale image recovery can be seen in Fig. S1(a), (b). We can see that more phase levels will lead to high image recovery quality. But when phase levels are greater than 8, the growth is not obvious.

For the diffraction efficiency of the multiple phase levels with the phase of $\phi_{l}(l=0,1,\ldots,L-1)$. $L$ is number of the phase levels. The $m$ level diffraction efficiency of the multistep metasurface can be expressed by the following equation

$\eta_{m}=\frac{1}{L^{2}}sinc^{2}\left( \frac{m}{L} \right)\left[ C^{2}\left( m \right)+S^{2}(m) \right]$ (S4)

$C\left( m \right)=\sum_{l=0}^{L-1} cos(\phi_{l}-\frac{2\pi lm}{L})$ (S5)

$S\left( m \right)=\sum_{l=0}^{L-1} sin(\phi_{l}-\frac{2\pi lm}{L})$ (S6)

Based on Eq. (S4), (S5), (S6), if $\phi_{l}=\frac{2\pi l}{L}$, the diffraction efficiency is shown in Fig.S2 (c). More phase levels can obtain higher efficiency. As we know, the scaling of the phase will only change the efficiency but not change the information of the hologram. So Fig. 2 (c) shows the efficiency of the phase $\phi_{l}=s\frac{2\pi l}{L}$, $s$ is the phase scaling coefficient.

**Section 2. Hologram image pre-compensation**


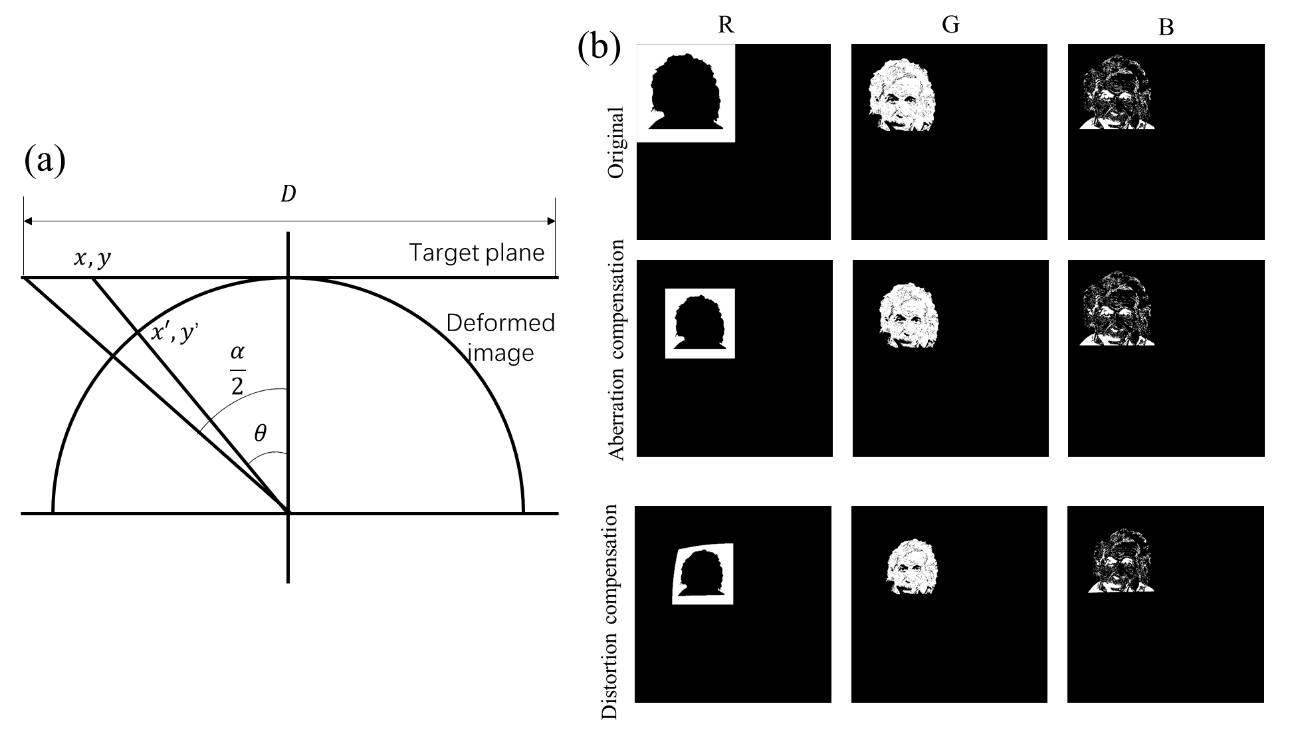


**Figure S2. Hologram image pre-compensation.** (a) Schematic of pre-compensation for distortion elimination. (b) The original images and pre-compensated images of trichromatic portrait of Albert Einstein.

From Eq. (S2), we can see that the $\left| e^{ik\left( z+\frac{\xi^{2}+\zeta^{2}}{2z} \right)} \right|$=1. Therefore, when $\lambda z$ is a constant, the same E-field distribution will be achieved in the far field. So to ensure the same size on the same screen for R, G and B channels for color holography, the target hologram image projected by laser of $\lambda_{j}$ should be scaled by the ratio of $\frac{\lambda_{i}}{\lambda_{j}}$ to match the size of image projected by laser of $\lambda_{i}$in the screen to ensure the accurate color mixing.

The field of view (FOV) of the meta-hologram can be calculated by

$\alpha=2\arctan\frac{\lambda}{{2p}_{2}}$ (S7)

Where $p_{2}$ is the period of the metasurface. Therefore, for the meta-hologram in our device, the maximum projection angle is 76.7$^{\circ}$, 67.2$^{\circ}$ and 58.7$^{\circ}$ for 633 nm, 532 nm and 450 nm wavelength. Due to the large FOV, the distortion of the hologram projection will be obvious in the far field.

To eliminate the distortion, the target hologram image can apply a reverse deformation. As Fig. S2 (a) shown, the coordinate of the deformed image can be expressed by

$x'=x/\sqrt{1+\frac{{(x}^{2}+y^{2})}{\left( \frac{D}{2} \right)^{2}}\tan^{2} \frac{\alpha}{2}}$ (S8)

$y'=y/\sqrt{1+\frac{{(x}^{2}+y^{2})}{\left( \frac{D}{2} \right)^{2}}\tan^{2} \frac{\alpha}{2}}$ (S9)

where $x, y$ is the coordinate of the original target image. $D$ is the size of the target image. Because the deformation is a function of FOV, the compensation for each wavelength is different.

To get a color hologram image in the far field, the target image will first be separated to three primary color components. Then the components will be scaled for each wavelength. Finally, the scaled components will be deformed base on Eq. (S8), (S9). Fig. S2 shows the original images and the pre-compensated images of trichromatic portrait of Albert Einstein.

**Section 3. Color filter design**


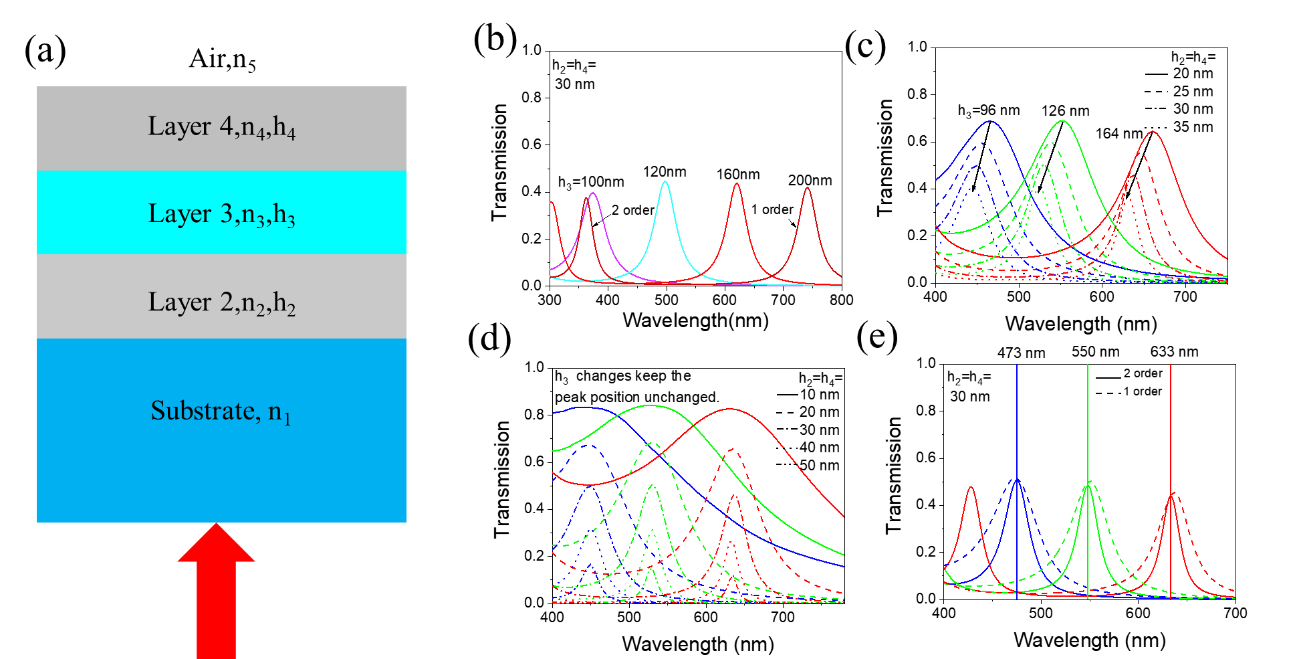


**Figure S3. Color filter design.** (a) Schematic of multi-layer interference model. (b) Transmission spectra of Ag/hydrogen silsesquioxane (HSQ)/Ag structure with varying HSQ thickness ($h_{3}$) and 30 nm-thick silver layer. (c) Transmission spectra with varying silver layer thickness ($h_{2}, h_{4}$) with fixed HSQ thickness for RGB channels. (d) Transmission spectra with varying silver layer thickness ($h_{2}, h_{4}$) and HSQ thickness($h_{3}$) keeping the peak position unchanged. (e) Comparison of 1-order and 2-order spectra.

As shown in Fig.S3 (a), for the normal incidence from substrate through the multi-layer film. The total transmission and reflection coefficients of layer 2 can be calculated by the following equation according to the multi-layer film interference theory.

${t_{2}}=\frac{t_{32}t_{21}e^{i\delta_{2}}}{1+r_{23}r_{12}e^{i\delta_{2}}},r_{2}=r_{12}+\frac{{r_{3}t_{12}t_{21}}e^{i{2\delta}_{2}}}{1+r_{12}r_{3}e^{i{2\delta}_{2}}}$ (S10)

The same procedure can be easily adapted to obtain the total transmission and reflection coefficients for the other layers.

${t_{3}}=\frac{t_{43}t_{2}e^{i\delta_{3}}}{1+r_{34}r_{2}e^{i\delta_{3}}},r_{3}=r_{23}+\frac{{r_{4}t_{23}t_{32}}e^{i{2\delta}_{3}}}{1+r_{23}r_{4}e^{i{2\delta}_{3}}}$ (S11)

${t_{4}}=\frac{t_{54}t_{3}e^{i\delta_{4}}}{1+r_{45}r_{3}e^{i\delta_{4}}},r_{4}=r_{34}+\frac{{r_{34}t_{23}t_{32}}e^{i{2\delta}_{4}}}{1+r_{34}r_{45}e^{i{2\delta}_{4}}}$ (S12)

Finally, the transmittance of entire stack structure can be obtained by

$T={\frac{\hat{n}_{1}}{\hat{n}_{5}}\text{ }\left| t_{4} \right|}^{2}$ (S13)

where

$\delta_{i}=\frac{2\pi\hat{n}_{i}h_{i}}{\lambda},{r_{ij}}=\frac{\hat{n}_{i}-\hat{n}_{j}}{\hat{n}_{i}+\hat{n}_{j}},{t_{ij}}=\frac{{2\hat{n}}_{i}}{\hat{n}_{i}+\hat{n}_{j}}$ (S14)

$\hat{n}_{i}$ is the complex refractive index of layer $i$. By calculating the reflection and transmission coefficient follow this procedure: $r_{4}\to r_{3}\to r_{2}\to t_{2}\to t_{3}\to t_{4}$. Then the transmittance of the stack structure can be calculated by Eq. (S13).

Here, the stack structure is same as the structure applied in the 3D-integrated metasurfaces device. The substrate is SiO_2_, layer 2 and layer 4 are silver and the layer 3 is SiO_x_ exposed by HSQ. Fig.S3 (b) shows transmission spectra with varied HSQ thickness and 30 nm-thick layer. We can see that a relative narrow spectrum of different peak positions can be achieved by altering the thickness of the HSQ layer. In addition, thicker dielectric layers lead to higher order peaks in the visible range. The transmission spectra with varying silver layer thickness ($h_{2}, h_{4}$) with fixed HSQ thickness for RGB channels in Fig.S3 (c) shows a thicker silver layer will cause a blue shift. By varying silver layer thickness ($h_{2}, h_{4}$) and HSQ thickness($h_{3}$) at the same time to keep the peak position unchanged, Fig.S3 (d) shows that a thicker silver layer narrows the spectrum to suppress crosstalk, but the efficiency is also reduced. To achieve smaller crosstalk, we can take advantage of narrower higher order peaks. By selecting the appropriate wavelength, the crosstalk of the 2-order peak shown in the Fig.S3 (e) is significantly smaller than the crosstalk of the 1-order peak. The 2-order peak average crosstalk is 3.5%, which is about half smaller than the 1-order peak of 6.5%.

**Section 4. Fabrication of the samples.**


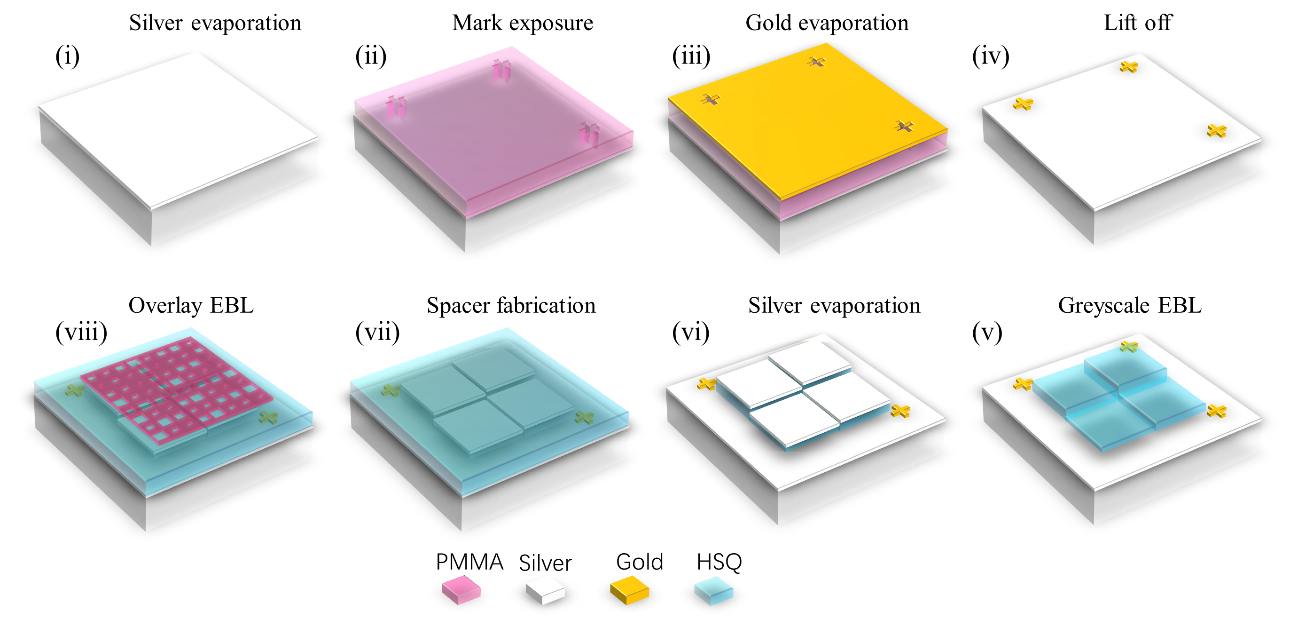


**Figure S4. Fabrication process of 3D-integrated metasurface.** (i) A 30-nm-thick silver layer was deposited on SiO_2_ substrate. (ii) EBL of the marks for overlay process. (iii) A 30 nm-thick gold was evaporated. (iv) Lift-off process to obtain gold marks. (v) Greyscale lithography on a 200 nm HSQ. (vi) Deposition of a 30-nm-thick silver film top layer to form stepwise FP cavity resonators. (vii) A HSQ spacer was spin-coated to protect the color filter microarray. (viii) Overlay EBL process to define binary nanostructures.

The fabrication process of 3D-integrated metasurface device is shown in Fig. S4. First, the SiO_2_ substrate was evaporated with a silver layer using thermal evaporation with rate of 0.3 $\dot{\text{A}}\text{/s}$. Then 300 nm poly methyl methacrylate (PMMA) electron-beam resist layer was spin-coated. The sample was then exposed through electron-beam lithography (EBL) with 30-kV voltage and a beam current of 272 pA. The exposure dose of 300 $\text{μC/}\text{cm}^{\text{2}}$ was used to expose the marks for overlay process. Then a 30-nm-thick gold was evaporated. The lift-off process of the sample was done in a solution of N-Methyl pyrrolidone (NMP). After the fabrication of gold marks, 200 nm hydrogen silsesquioxane (HSQ) was spin-coated on the sample as a negative-tone resist. By carefully controlling the exposure doses, we could control the thickness of patterned HSQ resist after the development. The exposure dose was determined according to the color microprint image and the palette database in section 5. Subsequently, a silver film top layer was evaporated again to form the stepwise FP cavity resonators. The spacer was then fabricated by exposing a 150 nm HSQ to protect the color filters. Finally, a layer of 400 nm PMMA was spin-coated on the sample and then exposed with overlay EBL process to define the binary nanostructures.

**Section 5. Color palette characterization**


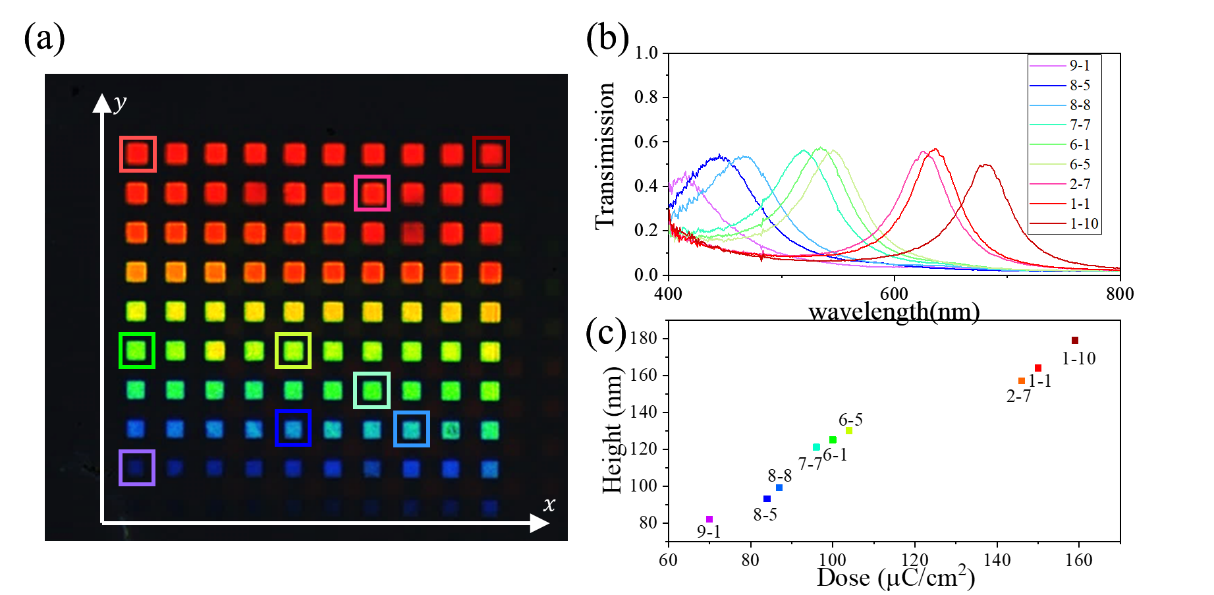


**Figure S5. Color palette and its characterization.** (a) The palette with different exposure dose. (b) The measured transmission spectra for selected blocks in (a). (c) The measured height as function of the exposure dose.

The color palette shown in the Fig.S5 (a) was fabricated to determine the greyscale lithography dose for different dielectric layer heights and resonance wavelengths. In the palette, the size of each block is 10 $\mu m$. The exposure dose is increased along the $x$ and $y$ axis. Figure S5 (b) is the measured transmission spectra of selected color block shown in Fig.S5 (a). The resonance wavelengths span the visible light range. Fig.S5 (c) shows the measured thickness of the dielectric layer as a function of the exposure dose, indicating that the height varied from 80 nm to 180 nm when the exposure dose increased from 60 $\text{μC/}\text{cm}^{\text{2}}$ to 160 $\text{μC/}\text{cm}^{\text{2}}$. The palette is the important database for designing of the color microprint.

**Section 6. Impact of upper layers on color filters**

**
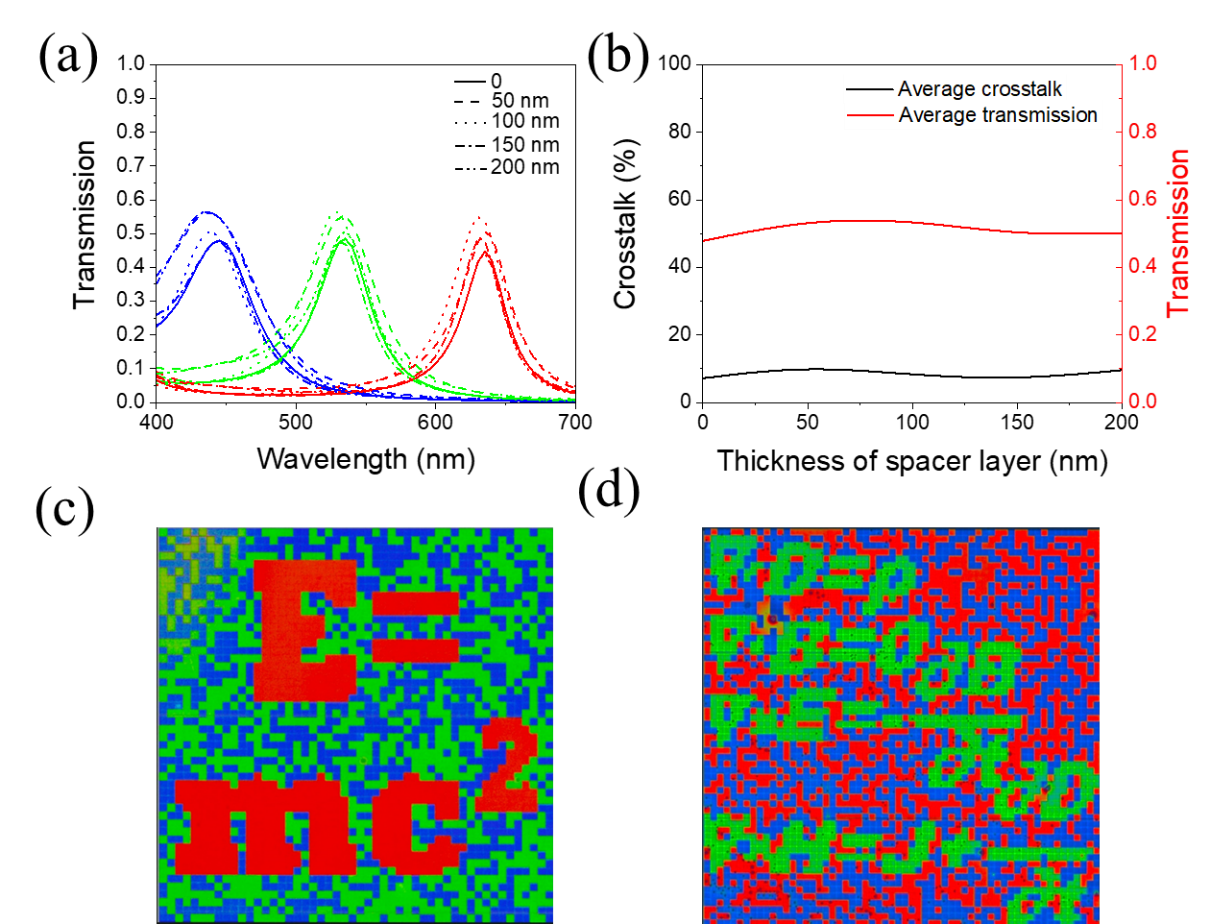
**

**Figure S6. Impact of upper layers on color filters.** (a) Transmission spectra with different thickness of spacer layer of RGB channels. (b) Effect of spacer on the crosstalk and transmission. (c)(d) The figures of trichromatic microprints without spacer layer and hologram metasurface on it.

Fig. S6 shows the impact of upper layers on color filters. Because the spacer layer and PMMA have similar refractive index, we assumed the metasurface to be a continuous film and calculated the total effect of the upper layers. We can see that the upper layers will lead to a slight blue shift of the spectra and an increase of transmission efficiency. In Fig. S6 (b), we calculated the average crosstalk and transmission efficiency with varying thickness of upper layers. It shows that the upper layers have a limited effect on the crosstalk and will increase the transmission efficiency. The impact is cyclical because the upper layers acts as an antireflection layer and its maximum antireflection effect is proportional to wavelength.

To further study the impact of upper layers, the figures of trichromatic microprints trichromatic microprint without spacer layer and hologram metasurface on it are shown in Fig. S6 (c), (d). Comparing to the results with upper layers in the main text, we can see that the contour of the holographic structures have a slight influence on the purity of the color, but the influence on the expression of microprint information is negligible. Moreover, through the above calculation results, the influence of slight color shift on crosstalk is also limited.

**Section 7. Hologram characterization setup**


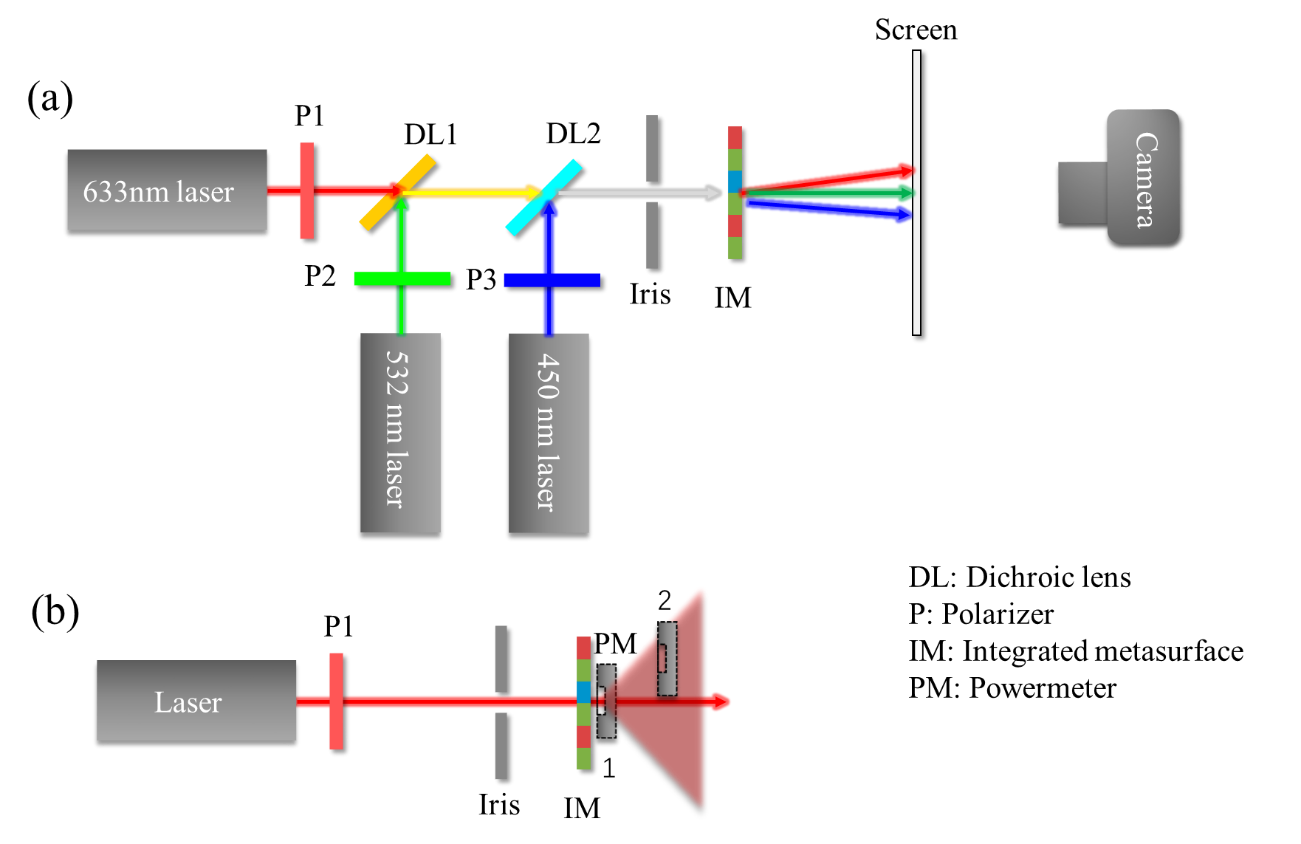


**Figure S7. Hologram characterization setup.** (a) Hologram image capture setup. (b) Diffraction efficiency characterization setup.

The hologram characterization setup is shown in Figure S7. The three laser diodes emitting at 450 nm (Thorlabs CPS450), 532 nm (Thorlabs CPS532), and 633 nm (Thorlabs CPS633S) were exploited to generate R, G and B channels. Due to the polarization independent property of the 3D-integrated metasurfaces, the polarizers were used to manipulate the source power incident on the device. Then two dichroic lens (DMLP567T, DMLP490T) was used to combine the three lasers. An iris was applied as a spatial filter to modify the spot of laser beam. Then the laser illuminated on the 3D-integrated metasurfaces. The hologram was then projected on a screen and captured by a SLR camera. To characterize the diffraction efficiency for each wavelength, we put an optical power meter in position 1 and position 2 as Fig. S7 (b) shown to measure the transmission power and the diffraction power of the hologram image.

**Section 8. Spectra of “four color theorem” painting**

**Figure S8. Spectra of “four color theorem” painting.** Navy and blue are chose for B channel color filter. Green is chose for G channel color filter. Red is chose for R channel color filter.

**Section 9. Characterization of devices with 26-nm-thick silver layer**


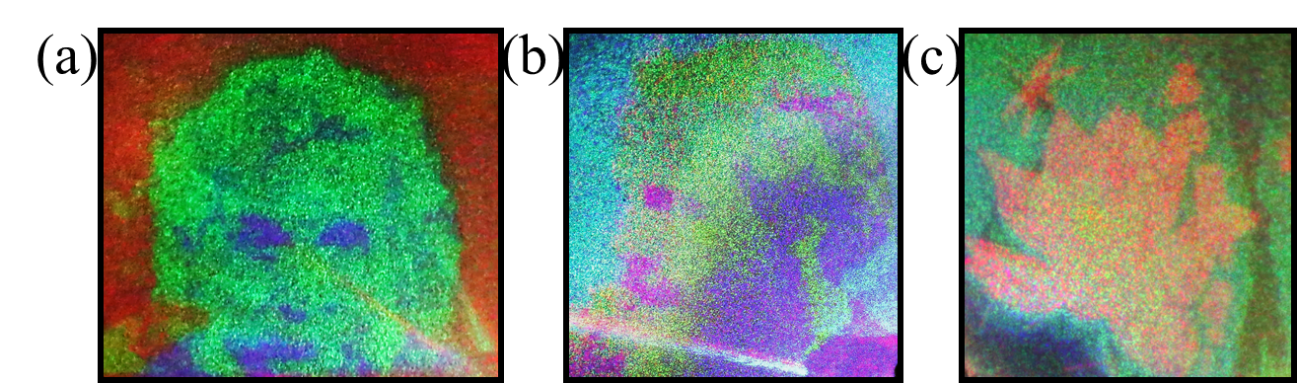


**Figure S9. 26-nm-thick silver layer devices characterization.** Experimental results of hologram images: (a) Trichromatic portrait of Albert Einstein. (b) Mixed color portrait of Jams Clerk Maxwell. (c) “Chinese painting of lotus”

**Section 10. Comparative holography experiment without color filter microarray**


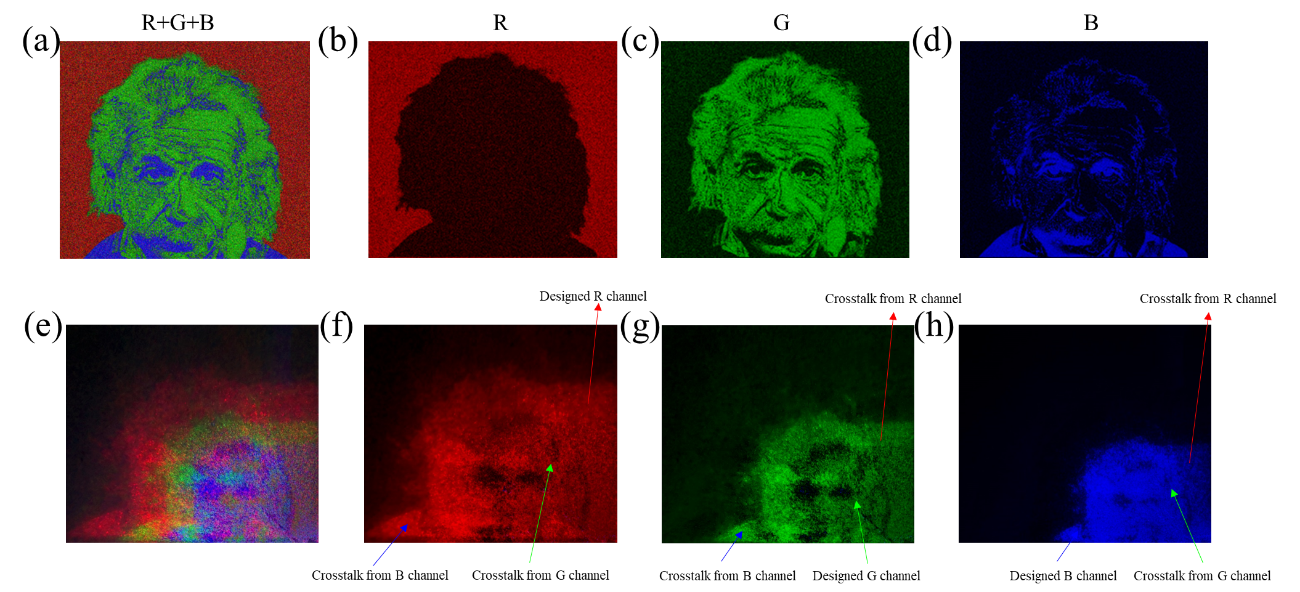


**Figure S10. Designed holography image and comparative holography experiment without color filter microarray.** (a) Designed trichromatic portrait of Albert Einstein image and its (b) R, (c) G, (d) B components. Comparative holography experiment without color filter microarray (e) R+G+B, (f) R, (g) G, (h) B components.
